# Reconstruction of Auto-Tissue-Engineered Lamellar Cornea by Dynamic Culture for Transplantation: A Rabbit Model

**DOI:** 10.1371/journal.pone.0093012

**Published:** 2014-04-04

**Authors:** Zheng Wu, Qiang Zhou, Haoyun Duan, Xiaoran Wang, Jianhui Xiao, Hucheng Duan, Naiyang Li, Chaoyang Li, Pengxia Wan, Ying Liu, Yiyue Song, Chenjing Zhou, Zheqian Huang, Zhichong Wang

**Affiliations:** State Key Laboratory of Ophthalmology, Zhongshan Ophthalmic Center, Sun Yat-sen University, Guangzhou, China; University of Reading, United Kingdom

## Abstract

To construct an auto-tissue-engineered lamellar cornea (ATELC) for transplantation, based on acellular porcine corneal stroma and autologous corneal limbal explants, a dynamic culture process, which composed of a submersion culture, a perfusion culture and a dynamic air-liquid interface culture, was performed using appropriate parameters. The results showed that the ATELC-Dynamic possessed histological structure and DNA content that were similar to native lamellar cornea (NLC, p>0.05). Compared to NLC, the protein contents of zonula occludens-1, desmocollin-2 and integrin β4 in ATELC-Dynamic reached 93%, 89% and 73%, respectively. The basal cells of ATELC-Dynamic showed a better differentiation phenotype (K3^−^, P63^+^, ABCG2^+^) compared with that of ATELC in static air-lift culture (ATELC-Static, K3^+^, P63^−^, ABCG2^−^). Accordingly, the cell-cloning efficiency of ATELC-Dynamic (9.72±3.5%) was significantly higher than that of ATELC-Static (2.13±1.46%, p<0.05). The levels of trans-epithelial electrical resistance, light transmittance and areal modulus variation in ATELC-Dynamic all reached those of NLC (p>0.05). Rabbit lamellar keratoplasty showed that the barrier function of ATELC-Dynamic was intact, and there were no signs of epithelial shedding or neovascularization. Furthermore, the ATELC-Dynamic group had similar optical properties and wound healing processes compared with the NLC group. Thus, the sequential dynamic culture process that was designed according to corneal physiological characteristics could successfully reconstruct an auto-lamellar cornea with favorable morphological characteristics and satisfactory physiological function.

## Introduction

More than 10 million patients worldwide are in need of corneal transplants. However, the shortage of corneal donors ultimately means that 1.5–2.0 million patients annually have untreated corneal blindness [1]. In theory, tissue engineering could remedy this problem by reconstructing a functional corneal equivalent in vitro. After an encouraging investigation by Prof. Griffith in 1999 [2], various types of corneal equivalents have been reconstructed in vitro.

For epithelial stem cell efficiency, cell sheets without scaffold or with a thin carrier are effective [3]–[9]. For corneal stroma injury, a thicker corneal scaffold of decellularized stroma provides a favorable healing outcome [10], [11], and a scaffold made from recombinant human collagen type III without seeding cells has been conducted successfully in a clinical trial [12]. However, just as Prof. Griffith has said, it is unlikely that there will be a single ‘‘one-size-fits-all’’ corneal substitute for all indications [13]. When both the corneal epithelium and deep stroma were damaged simultaneously, a functional lamellar cornea properly constructed from corneal epithelial cells with thicker scaffold was required for treatment [14]. Static air-liquid interface culture, which was first introduced by Minami in 1993 [15], has been applied widely to induce epithelial differentiation during the corneal reconstruction process. Using this process, 2–6 layers of stratified epithelium could be formed on different scaffolds [15]–[22], adhesive molecules and cell-cell junctions have been verified by immunofluorescence staining [17] and transmission electron microscopy [17]–[19]. The classical static culture method could promote epithelium growth and produce a favorable morphological outcome on amniotic membrane [23], compressed collagen [18], silk fibroin [24], corneal stromal lamella discs [25], and so on.

In our previous reports, acellular porcine corneal stroma (APCS) was prepared as a scaffold with favorable biocompatibility, adequate biomechanical intension and high transparency [11], [26], [27]. The limbal region retained the original niche of limbal stem cells, which was advantageous for the proliferation of corneal epithelium [28]. But this thicker scaffold (850 μm) would absorb water continuously. If scaffold edema in culture medium hindered the mass transfer of constructed epithelium would be questioned. And it remains unknown whether the biological properties of a reconstructed lamellar cornea, including epithelial viability, proliferation, adhesion, and differentiation, could coordinate precisely with each other and be adapted to a viable transplantation. So in this study, to reconstruct an auto lamellar cornea using APCS scaffold for viable transplantation, we investigated the feasibility of static air-lift culture compared with dynamic culture process, which was composed of a submersion culture, a perfusion culture and a dynamic air-liquid interface culture.

## Materials and Methods

### Ethics Statement and Animals

All of the procedures in this experiment were performed in accordance with the ARVO Statement for the Use of Animals in Ophthalmic and Vision Research and were approved by the animal ethics committee of Zhongshan Ophthalmic Center, Sun Yat-sen University, China.

Whole porcine eyes (Yorkshire swine, either gender, 6 months, 110–125 kg) were obtained within 1–3 h postmortem, and subjected to decellularization procedure within 1 h of receival. The eyes selected for the study had integral corneal surface with a horizontal corneal diameter of 12–14 mm and initial corneal thickness of 800–900 mm, as measured by an ultrasound pachymeter (SP100, Tomey, Japan). Young adult New Zealand white rabbits of either gender aged 10 weeks and weighed 2–3 kg, were used as animal transplant models.

### Preparation of APCS

APCS were prepared as previously described [11] **.** Briefly, the native porcine corneas were soaked in deionized water for 1 h at 10°C. Next, the corneas were immersed in a bicarbonate-mixed salt solution containing phospholipase A_2_ (200 U/ml; Sigma) and 0.5% (w/v) sodium deoxycholate (Sigma) for 6 h at 37°C. After rinsing with the bicarbonate-mixed salt solution 3 times for 10 min each, the samples were immersed in a bicarbonate-mixed salt solution containing only phospholipase A_2_ for 2 h at 37°C. All steps were conducted with continuous shaking in a thermostat-controlled water bath. The prepared APCS were dried in a thermostatic drier at 37°C for 72 h to a constant weight. Finally, the corneas were sealed into a sterile plastic envelope, sterilized by γ-irradiation (25 kGy), and stored at 4°C before use.

### The Major Components of Basement Membrane in APCS

The 10 mm cryostat sections of APCS (n = 4) were used for immunofluorescence staining. The primary antibodies were collagen I (1∶100, Thermo Scientific), collagen IV (1∶50, Birmingham), laminin (1∶100, Thermo Scientific), fibronectin (1∶100, Abcam) and collagen VII (1∶50, Abcam). The secondary antibodies were Alexa Fluor 594 IgG (1∶100, Gentaur) and Alexa Fluor 488 IgG (1∶100, Invitrogen). A DNA-specific dye, Hoechst 33258 (1∶2000, Invitrogen), was used to detect nuclei, and all sections were viewed using a laser scanning confocal microscope (LSM 510 META, Carl Zeiss, Germany). Furthermore, the ultrastructures of 4 APCS preparations were examined using transmission electron microscopy (TEM, H600, Hitachi, Japan) and scanning electron microscopy (SEM, JSM-6330F, JEOL, Japan).

### Dynamic Culture Process

**Figure 1:**
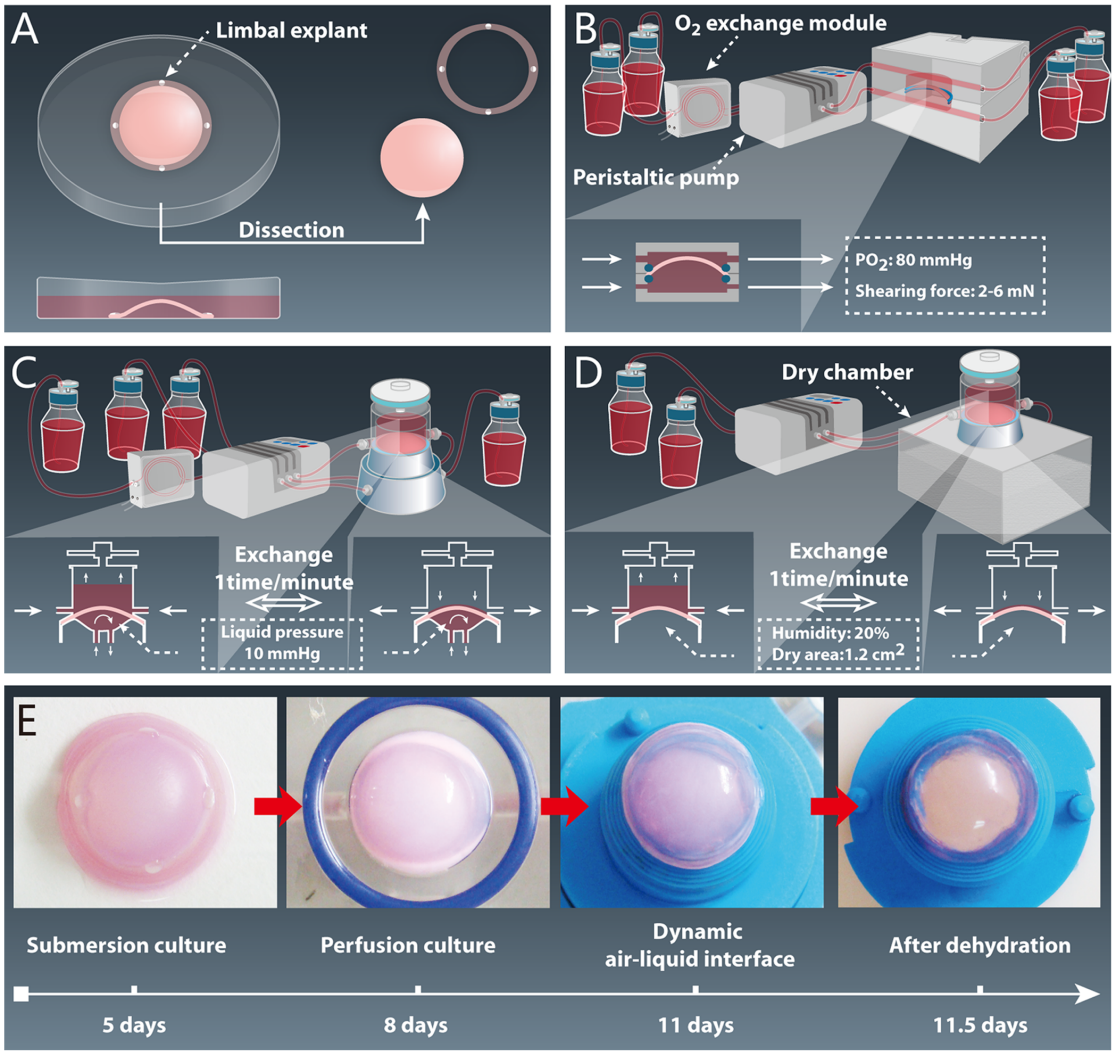
The construction process of auto-tissue-engineered lamellar cornea. (A) Submersion culture (5 days). (B) Perfusion culture (3 days). (C) Dynamic air-liquid interface culture (with perfusion culture on the bottom surface, 3 days). (D) Dynamic air-liquid interface culture (with a dry chamber on the bottom surface, 12 hours). (E) Macroscopic view of ATELC-Dynamic in each stage of the construction process.

In Fig. 1A, submersion culture, 100 μl of DMEM supplemented with 200 ng EGF and 400 ng KGF was absorbed by the posterior surface of the APCS for 24 hours at an incubation temperature of 37°C. Then, 4 corneal limbal explants (100 μm thickness, 1 mm diameter) were obtained from the left eyes of the rabbits, placed on the limbal part of APCS with the epithelial side down, and cultured in 10 ml of medium (the composition of which came from our previous study [29], [30] at 37°C in a 5% CO_2_ incubator for 5 days. The concentrations of EGF and KGF released by the APCS were 10 ng/ml and 20 ng/ml, respectively.

In Fig. 1B, perfusion culture, the samples that had been dissected with a 14-mm trephine were placed into a perfusion culture system that connected a peristaltic pump (IPC-N Ismatec, Glattbruch-Zurich, Switzerland) with equipment for O_2_ exchange (Minucells, Regensburg, Germany). There, culture medium (1000 ml) with high oxygen pressure (80 mmHg) was applied to both sides of the samples along a gradient of increasing fluid shearing force (1^st^ day: 2 mN, 2^nd^ day: 4 mN, 3^rd^ day: 6 mN).

Then, the sample was fixed into the dynamic air-liquid interface culture. On the upper surface, the interval of air-liquid exchange was set to 1 exchange per minute, and oxygen pressure from the atmosphere reached 155 mmHg. On the bottom surface the perfusion culture (500 ml medium) was maintained, but the perfusion pressure was increased to 10 mmHg (Fig. 1C). After 3 days, the perfusion culture on the bottom surface was replaced with a dry chamber (37°C, humidity: 20%, dry area: 1.2 cm^2^) for12 hours to recover 80% of the water content (Fig. 1D).

To estimate the morphological characteristics of the auto-tissue-engineered lamellar cornea using dynamic culture (ATELC-Dynamic), the central part of the native lamellar corneas (NLC) from rabbits served as a positive control group. Specimens that were subjected to the same submersion culture (stage 1) and then constructed using a static air-liquid interface culture (15) for 6.5 days, served as a negative control group (ATELC-Static). Specimens from these 3 groups were observed using Hematoxylin and Eosin (H&E) staining (n = 4), TEM (n = 4), SEM (n = 4) and quantitative DNA analysis (n = 10, Sigma).

### The Differentiation Phenotype, Proliferative Activity and Adhesion Property Assay

The differentiation phenotype was assessed with immunofluorescence staining (n = 4) for K3 (1∶100, Millipore), P63 (1∶50, Millipore) and ABCG2 (1∶50, Millipore). Proliferative activity was measured via colony-forming efficiency (n = 10), as previously described [31]. Briefly, the epithelium of each specimen was trypsinized and planted at a density of 500 cells per 60 mm in dishes that had been preseeded with 3T3 fibroblast feeder layers. Colony-forming efficiency was calculated on day 6. Cell junction-related proteins zonula occludens-1 (1∶50, Invitrogen), desmocollin 2 (1∶100, Abcam), and integrin β4 (1∶50, Abcam) were detected using immunofluorescence staining (n = 4). Then, the contents of these proteins were quantitated using ELISA assays according to the manufacturer’s recommendations (n = 10, Cusabio Biotech). The ultrastructures of cell junctions in each group were observed using TEM (n = 4).

### Trans-epithelial Electrical Resistance (TER)

To evaluate epithelial barrier function, trans-epithelial electrical resistance (n = 10) was measured using an Epithelial Volt-ohm meter and Ag/AgCl electrodes (EVOMX, World Precision Instruments, Sarasota, FL). The trans-epithelial electrical resistance of each sample was measured before (TER1) and after (TER2) the epithelia were removed by exposure to 0.02 M EDTA for 1 hour. Finally, the TER of each sample was calculated as follows: TER = TER1-TER2.

### Light Transmittance Assay

The samples (n = 10) were directly fixed into the specimen chamber of a UV–VIS recording spectrophotometer (UV-2501, SHIMADZU, Japan), and light transmittance was measured over a range of light wavelengths (300∼800 nm) in intervals of 10 nm [11].

### Stress–strain Assay

The stress–strain assay was performed to assess biomechanical characteristics, as previously described [11]. An automatic syringe pump (SP-500, JMS, Singapore) was attached to one inlet of an artificial interior chamber (K20–2125, Katena, USA), while the other inlet was connected to a pressure gauge. Each sample (n = 10) was in turn attached to the artificial interior chamber, and the system was kept airtight. Samples were tested by a stress–strain assay in the 10–60 mm Hg pressure range. A 2 mm diameter trephine tipped with Alcian blue solution was touched lightly to the central zone of the cornea, forming a round area on the surface. The round area on the surface of the sample was photographed as the primary area (S_0_) at 10 mmHg pressure. Pressure was increased gradually to 20, 30, 40, 50 and 60 mmHg. Thus, five pressure differences (P’) of 10, 20, 30, 40 and 50 mm Hg were respectively obtained, and the new areas of staining (St) were also photographed. S_0_ and St in the acquired photographs were measured by image analysis software (Image-Pro 10.0). The strain (γ) of an area was calculated using the formula, γ = (S_t_–S_0_)/S_0_. The areal modulus was calculated using the formula, areal modulus = P’/γ.

### Rabbit Lamellar Keratoplasty

ATELC-Dynamic, ATELC-Static, and NLC were labeled using DiO (5 μg/ml, Invitrogen) for 30 minutes at 37°C and were implanted into the right corneas of rabbits by routine lamellar keratoplasty (n = 14, 100 μm thickness, 6.25 mm diameter). Under general anesthesia (using intramuscular injection of ketamine hydrochloride, 25 mg/kg), a 100 μm deep circular incision 6 mm in diameter was made using a Barraquer trephine. A lamellar dissection was performed using an operating knife along a natural uniform stratum in the corneal stroma to remove host epithelium and anterior stroma. The ATELC-Dynamic, ATELC-Static, and NLC grafts were sutured into the recipient bed with eight interrupted 10–0 nylon sutures. Tobramycin–dexamethasone eye ointment was applied 3 times daily for 7 days after LKP. After transplantation, sodium fluorescein staining was conducted to assess epithelial integrity, and slit lamp examination was performed to assess for optical clarity of the cornea, corneal neovascularization, and degradation of corneal grafts. On postoperative day 7, 4 rabbits from each group were euthanized, and their corneas were prepared for immunofluorescence assay with DiO to assess the proliferation and migration of epithelial cells. On postoperative day 20, 10 rabbits were euthanized, and corneal specimens from those rabbits were evaluated using light transmittance assays and immunofluorescence staining for collagen III (1∶50, Acris).

### Statistical Analysis

Values are shown as the mean ± standard deviation. SPSS 11.0 software was used for statistical analysis, and the differences between the groups were compared using one-way ANOVA. Statistical significance was defined as p<0.05.

## Results

### The Major Components of Basement Membrane in APCS

**Figure 2:**
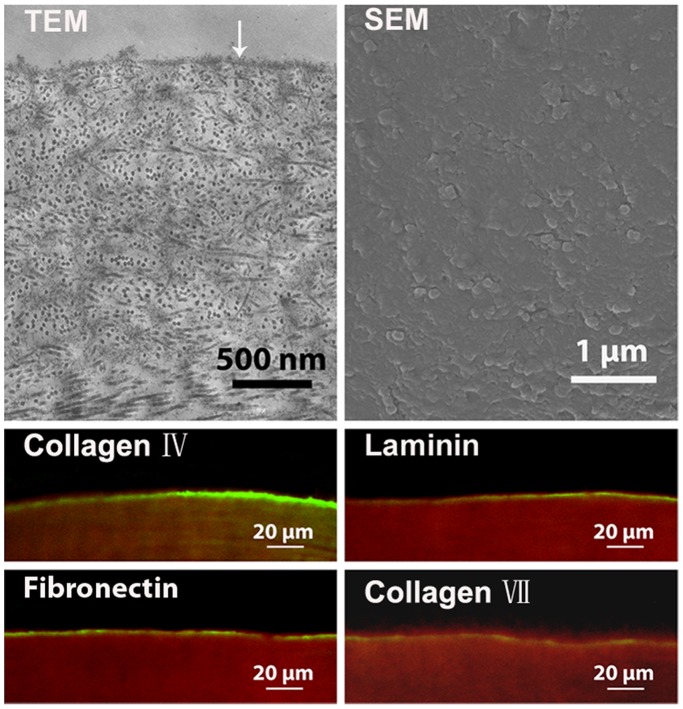
The major components of the basement membrane in APCS (↓: basement membrane components, green: collagen IV, laminin, fibronectin and collagen VII, red: collagen I).

TEM and SEM images showed that the basement membrane existed and was distributed uniformly on the anterior surface of APCS. Immunofluorescence staining identified the major components of the basement membrane, including collagen IV, laminin, fibronectin and collagen VII (Fig. 2, n = 4).

### Changes in Histomorphology and DNA Content during Construction

**Figure 3:**
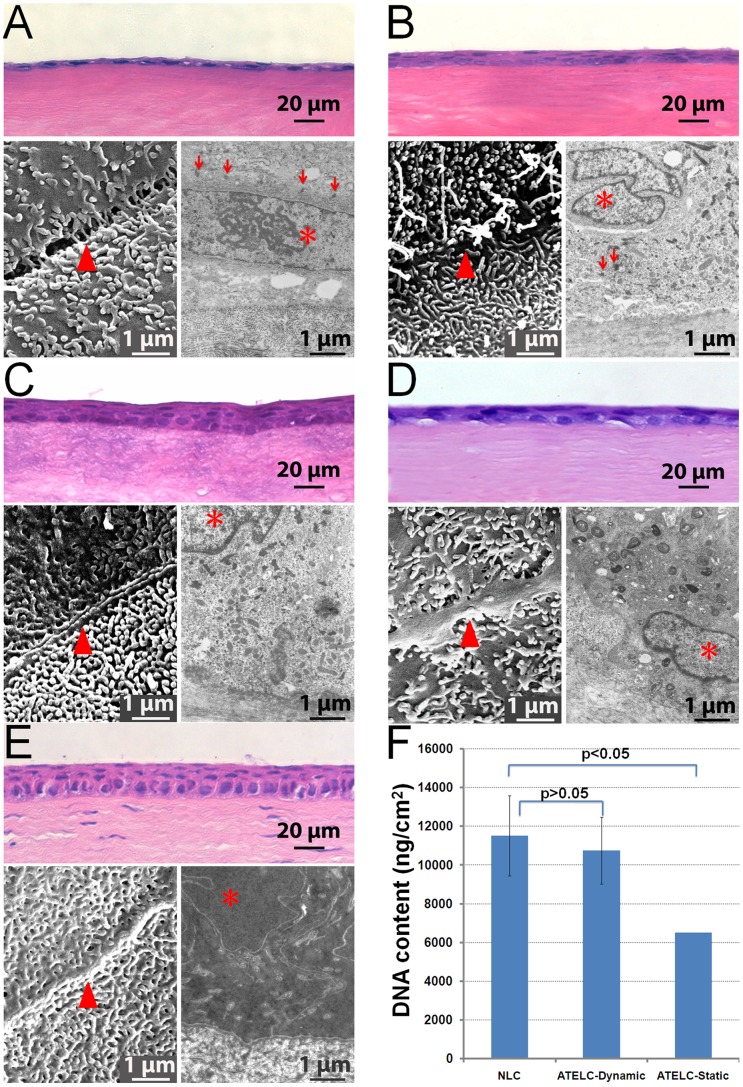
The changes in histomorphology and DNA content during the construction process (▴: connection between neighbor cells, *: cellular nucleus,↓: desmosome). (A) ATELC-Dynamic, after submersion culture. (B) ATELC-Dynamic, after perfusion culture. (C) ATELC-Dynamic, after dynamic air-liquid interface culture. (D) Negative control: ATELC-Static. (E) Positive control: NLC. (F) DNA contents of the NLC, ATELC-Dynamic, and ATELC-Static groups.

After submersion culture (stage 1, Fig. 1A), the DNA content of samples was 3.363±1.025 μg/cm^2^ (n = 10). There were only 1–2 layers of squamous cells with flattened nuclei (Fig. 3A, H&E staining). The superficial cells formed connections with each other and gross microvilli emerged the basal cells underwent mitosis, and desmosome junctions were abundant. (Fig. 3A, SEM, and TEM).

When perfusion culture was performed (stage 2, Fig. 1B), the seeding cells proliferated rapidly, DNA content reached 8.102±1.489 μg/cm^2^ (n = 10). 3–4 layers of flattened epithelial cells formed a more compact epithelium, and the division of basal cells was still active and the microvilli became more mature than in stage 1 (Fig. 3B).

After dynamic air-liquid interface culture (stage 3, Fig. 1C, 1D), the constructed ATELC-Dynamic possessed a histological structure and ultrastructure similar to that of NLC, which consists of one layer of columnar basal cells and 3 layers of flattened superficial cells. The connections between neighboring superficial cells were tight, both the morphology and density of microvilli resembled those found in natural corneal epithelia. There were abundant chondrosomes in cytoplasm of the basal cells. (Fig. 3C, 3E). In addition, the DNA content of ATELC-Dynamic (10.752±2.073 μg/cm^2^, n = 10) was not significantly different from NLC (11.516±1.276 μg/cm^2^, n = 10, p>0.05).

For the negative control group, ATELC-Static also possessed 3–4 layers of epithelial cells, but the DNA content was only 6.502±1.721 μg/cm^2^ (n = 10, p<0.05, compared to ATELC-Dynamic). The image of the SEM (n = 4) showed the microvilli were fewer and connections between superficial cells were immature. Some swelling mitochondria and myelin figures were found in TEM image. (Fig. 3D).

### The Differentiation Phenotype, Proliferative Activity and Adhesion Property

**Figure 4:**
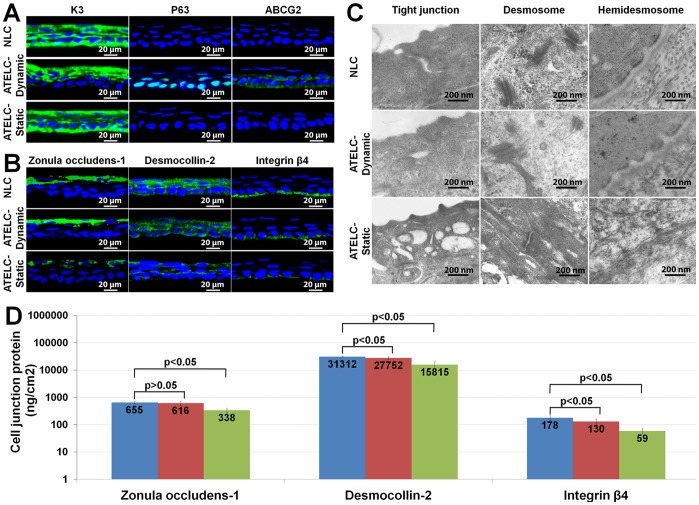
The differentiation phenotype and adhesion property of the epithelium in ATELC-Dynamic. (A) The expressions of K3, P63, ABCG2 proteins. (B) The expressions of zonula occludens-1, desmocollin-2 and integrin β4 proteins. (C) The ultrastructures of tight junction, desmosome junction and hemidesmosome junction. (D) ELISA assay of the zonula occludens-1, desmocollin-2 and integrin β4 proteins.

Fig. 4A showed that the basal layer of epithelial cells in the ATELC-Dynamic group was distinguished from that of the NLC and ATELC-Static groups (K3^+^, P63^−^, ABCG2^−^) by its phenotype: K3^−^, P63^+^, ABCG2^+^. Furthermore, the cell-cloning efficiency of the ATELC-Dynamic group (9.72±3.5%) was significantly higher than that of the NLC group (6.29±1.96%, n = 10, p<0.05) and the ATELC-Static group (2.13±1.46%, n = 10, p<0.05).

The adhesion property of the epithelium was shown in Fig. 4B. Zonula occludens-1, desmocollin-2 and integrin β4 proteins were all expressed in the epithelia of NLC, ATELC-Dynamic and ATELC-Static groups (n = 4). Quantitative assay revealed that the expression of these 3 proteins in the ATELC-Dynamic samples reached 93%, 89% and 73%, respectively, of NLC protein totals (Fig. 4D, n = 10). Meanwhile, the ultrastructures of the tight junctions, desmosome junctions and hemidesmosome junctions that formed in the ATELC-Dynamic group were also similar to those of native tissue (Fig. 4C, n = 4). However, in the ATELC-Static group, the ultrastructure of the cell junctions (Fig. 4C, n = 4) and the expression of cell junction proteins (Fig. 4D, n = 10, p<0.05) were significantly different from those of the ATELC-Dynamic group.

### Physiological Function in vitro

**Figure 5:**
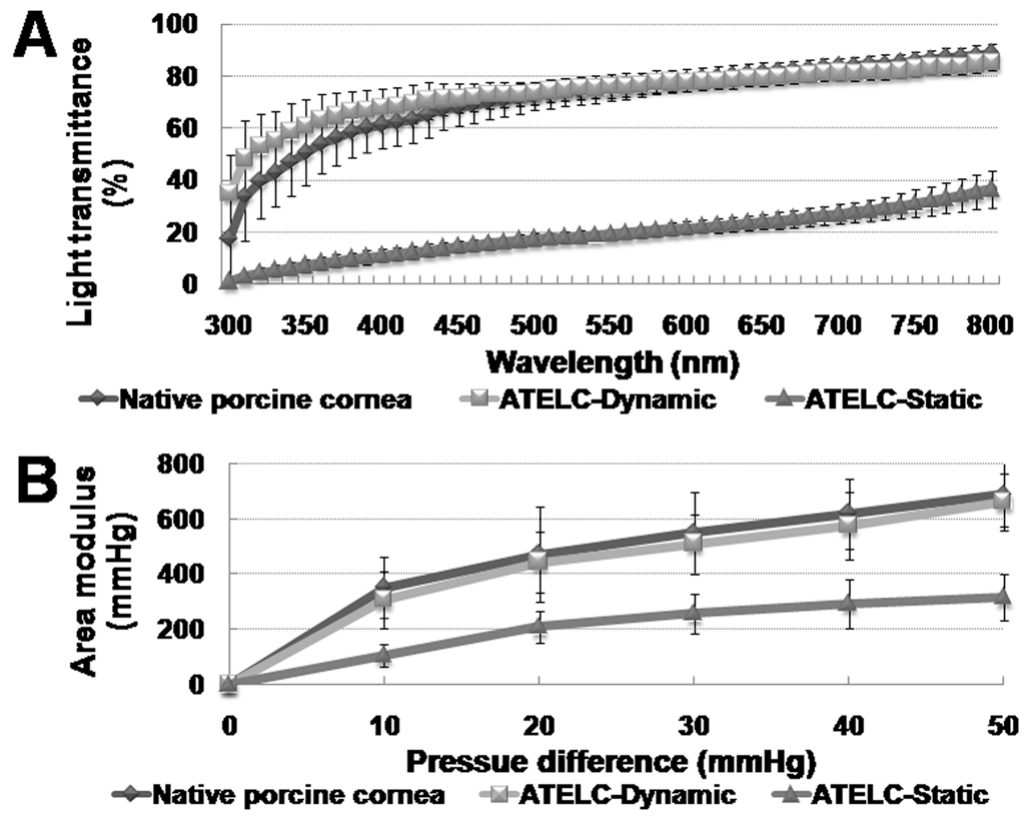
Physiological functions of ATELC-Dynamic in vitro. (A) Light transmittance over the 300–800 nm range of light wavelengths. (B) The areal modulus variation between 0 and 50 mmHg pressure differences.

The value of trans-epithelial electrical resistance in the ATELC-Dynamic group (72.0±14.7 Ω, n = 10) reached the level of the NLC samples (77.8±6.0 Ω, n = 10, p>0.05) and was significantly higher than that of the ATELC-Static groups (22.4±7.4 Ω, n = 10, p<0.05). Due to the 80% water content, the light transmittance of the ATELC-Dynamic group was similar to that of native porcine cornea (Fig. 5A, n = 10, p>0.05). Furthermore, the stress-strain assay revealed that the differences in areal modulus variation between the ATELC-Dynamic groups and native porcine cornea were not significant over a pressure difference that ranged from 0 to 50 mmHg (Fig. 5B, n = 10, p>0.05). However, in the ATELC-Static group, because of stoma edema, both light transmittance and areal modulus were significantly different compared to the ATELC-Dynamic and native porcine cornea groups.

### Rabbit Lamellar Keratoplasty

**Figure 6:**
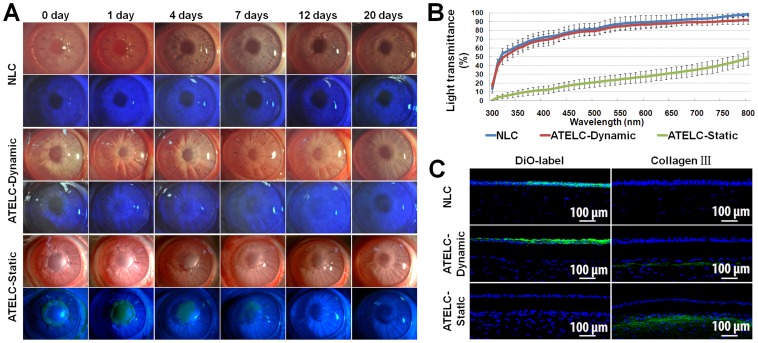
Physiological function of ATELC-Dynamic in a rabbit lamellar keratoplasty model. (A) Postoperative observation of lamellar keratoplasty. (B) The light transmittance of transplanted lamellar cornea over the wavelength range of 300–800 nm at 20 days. (C) DiO-labeled seeding cells at 7 days, and the expression of collagen III at 20 days.

Constructed ATELC-Dynamic displayed high transparency immediately after lamellar keratoplasty; the details of pupil boundary and iris texture were clearly revealed. Meanwhile, sodium fluorescein staining of animals in this group was negative (n = 14). Furthermore, corneal edema, neovascularization, and graft degradation were not observed at any time during the postoperative observation period (Fig. 6A). At 20 days post-operation, there was no significant difference in light transmittance of transplanted ATELC-Dynamic compared to transplanted NLC over a range of wavelengths from 300–800 nm (Fig. 6B, n = 10, p>0.05). In the ATELC-Static group, constructed epithelium was totally excoriated within 24 hours, and graft edema was aggravated until complete re-epithelialization by epithelial cells from the recipient was completed at day 12. The quantitative assays showed that the light transmittance of this group was far lower than that of the ATELC-Dynamic group or the NLC group at 20 days post-operation (Fig. 6B, n = 10, p<0.05). Similar to the NLC group, DiO-labeled seeding cells in the ATELC-Dynamic group could have been substituted with epithelial cells from the recipient through migration via concentric movement, and these two types of cells exhibited favorable confluence in images from postoperative day 7. There was no sign of rupture (Fig. 6C, n = 4). More collagen III protein was secreted in the ATELC-Static group than in the ATELC-Dynamic and NLC groups to repair the interval between the graft and the recipient bed (Fig. 6C).

## Discussion

Because the original architecture of a native matrix plays a critical role in guiding organ development, repair and physiological regeneration, decellularized matrix scaffolds may provide a promising alternative to synthetic scaffolds [32]. For functional reconstruction of lamellar cornea, the APCS scaffold prepared in a previous study showed a favorable foundation for use in optics and biomechanics [11]. The key focus of this study was how to select the seeding cells and design the construction process according the characteristics of the APCS scaffold and physiological properties of native lamellar cornea.

The biological components of APCS showed the following features: 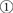
natural basement membrane components, including collagen IV, laminin, fibronectin and collagen VII, were retained in APCS, which were closely related to the biological functions of seeding cells (Fig. 2); 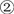
80% of the total glycosaminoglycans, which are capable of absorbing water and binding growth factors, were conserved after a decellularization process [11]. After it was supplemented with 200 ng EGF and 400 ng KGF, APCS exhibited a satisfactory sustained release of growth factors, thereby avoiding a sharp decrease of growth factor concentration due to the rapid proliferation of seeding cells. The presence of growth factors in the pericellular matrix is essential for the spatiotemporal coordination of cellular activities, including cell haptotaxis, which ensures proper tissue formation during wound healing [33].

To avoid the interference factor of immunologic rejection in transplant evaluation, the limbal explants from auto-corneas were selected as the source of seeding cells. This selection provided 4 additional advantages: 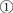
There are many poorly differentiated and highly proliferative epithelial stem cells in the basal part of the corneal limbus; 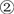
The sheet-like proliferation of epithelial cells in the explant culture could result in the formation of more mature cell-cell junctions(Fig. 3A) [34]; 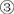
Utilizing limbus explants directly could save construction time and avoid cellular injury due to cell passage; 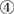
The safety of 180 degrees of limbal transplantation has been verified [35], and in this study, the 4 explants (1 mm each) only occupied 45 degrees of the total limbus.

After limbal explants were seeded on an APCS scaffold, the rapid proliferation and migration of epithelial cells was induced by the combination of a favorable extracellular matrix microenvironment and an optimal sustained release of EGF and KGF, implying that nutrition and oxygen levels were adequate at this stage (Fig. 3A). However, when the static air-liquid culture method was used, ATELC-Static groups produced 3–4 layers of epithelial cells but a DNA content of only 6.502±1.721 μg/cm^2^. Furthermore, the images of the ultrastructure (n = 4) showed some evidence of disordered energy metabolism in the basal cells (Fig. 3D). Scaffold edema (due to continuous water absorption of 850 μm APCS) may have hindered the mass transfer of constructed epithelium. It seemed that simple diffusion in a static air-liquid interface culture might not supply sufficient oxygen or nutrition for further proliferation and differentiation of seeding cells in this APCS scaffold.

As a result of this finding, a medium with a high oxygen pressure (80 mmHg) was applied to both sides of the constructed cornea through perfusion culture. The rapid proliferation of seeding cells was evidenced by an increase in DNA content (8.102±1.489 μg/cm^2^) and active cell division in the basal part of the constructed epithelium. A gradient of increasing fluid shearing force (2–6 mN) was applied to improve adhesion and ordered arrangement of seeding cells, in addition to the direct effect of promoting cell proliferation (Fig. 3B) [36]. Thus, a transition of epithelial cells from 2-dimensional growth to 3-dimensional growth on the APCS scaffold was implemented.

A dynamic air-liquid interface culture was used to promote the differentiation of the constructed epithelium. On the upper surface, the interval of air-liquid exchange was set to once per minute to mimic the blinking frequency of the rabbit, so that the constructed epithelium could utilize the higher oxygen content (155 mmHg) from the atmosphere and a continuous supply of nutrients. Furthermore, a continuous perfusion culture was applied to the bottom surface of the constructed tissue, but the perfusion pressure was increased to 10 mmHg to mimic the physiological intraocular pressure and to promote nutrition transfer through the APCS scaffold. Finally, a dry chamber was designed to dehydrate the constructed lamellar cornea during the last 12 hours of the construction process to prevent scaffold edema, which impairs optical property, biomechanical property and the wound healing process after transplantation [37]–[40].

Ultimately, the constructed ATELC-Dynamic had epithelial morphology and DNA content similar to NLC (Fig. 3C, 3E, 3F). The differentiation and proliferation features of the seeding cell source, the epithelium of the corneal limbus (K3^−^, P63^+^, ABCG2^+^), was retained by this dynamic culture process. The seeding cells on ATELC-Dynamic were obviously younger and had higher cell-cloning efficiency than those of NLC or ATELC-Static (Fig. 4A). Meanwhile, ATELC-Dynamic showed protein expression of zonula occludens-1, desmocollin-2 and integrin β4 that were 93%, 89% and 73%, respectively, of levels in NLC, indicating acceptable adhesion properties (Fig. 4D). The ultrastructures of three types of cell junctions in ATELC-Dynamic were similar to those in NLC (Fig. 4C). Therefore, ATELC-Dynamic could be constructed with adequate adhesion tension and moderate cell differentiation to produce favorable cell proliferation.

To withstand transplant operation and maintain tissue survival in vivo, reconstruction of functional engineered lamellar cornea should aim to maximize physiological functions. The tight alignment of a stratified epithelium and the abundance of cell junction expression contribute to favorable barrier-forming ability; the trans-epithelial electrical resistance of the corneal epithelium in ATELC-Dynamic was comparable to that of NLC (p>0.05). After dehydration in the last construction step, ATELC-Dynamic could recover its physiological water content and produce optical and biomechanical characteristics that are comparable with native lamellar cornea (Fig. 5, p>0.05).

In rabbit lamellar keratoplasty, ATELC-Dynamic was able to withstand the rabbit lamellar keratoplasty operation. During the entire observation period, the barrier function of ATELC-Dynamic was intact, without signs of epithelial shedding, corneal edema, or neovascularization (Fig. 6A). Furthermore, the ATELC-Dynamic group and the NLC group had similar corneal transparency (Fig. 6B). These results implied that the adhesion and proliferation properties of seeding epithelium in ATELC-Dynamic could withstand the intense change from laboratory culture to viable transplantation. When comparing the fates of seeding cells, it was found that the DiO-labeled epithelial cells of ATELC-Dynamic could be substituted with epithelial cells from the recipient bed through their migration via concentric movement, which was similar to the normal cell substitution that happened in the NLC-transplant group. These two types of cells exhibited favorable confluence; no sign of rupture between them was observed (Fig. 6C), suggesting that the cell function of seeding cells could coordinate with that of native cells to maintain the normal cell alternation. Furthermore, the ATELC-Dynamic group had a lower expression of collagen III (Fig. 6C), which was beneficial for long-term transparency of corneal tissue. Thus, in this transplantation model, ATELC-Dynamic had high optical transparency, stable biomechanics, and favorable epithelial barrier function.

## Conclusion

Using APCS as a scaffold and four 1-mm explants of auto corneal limbus as the source of seeding cells, the sequential dynamic culture process that was designed according to corneal physiological characteristics could successfully reconstruct an auto-lamellar cornea with favorable morphological characteristics and satisfactory physiological function. Although there is room in future studies to improve the dynamic culture parameters for endowing ATELC-Dynamic with further advantages, this study demonstrated that ATELC-Dynamic has potential for use in viable transplantation or clinical applications.
